# Acute Myeloid Leukemia Stem Cells in Minimal/Measurable Residual Disease Detection

**DOI:** 10.3390/cancers15102866

**Published:** 2023-05-22

**Authors:** Kritika Srinivasan Rajsri, Nainita Roy, Sohini Chakraborty

**Affiliations:** 1 Department of Pathology, New York University Grossman School of Medicine, New York, NY 10016, USA; ks3144@nyu.edu (K.S.R.); nainita.bhowmick@nyulangone.org (N.R.); 2 Department of Molecular Pathobiology, New York University College of Dentistry, New York, NY 10010, USA

**Keywords:** acute myeloid leukemia (AML), blasts, leukemia stem cells (LSCs), measurable/minimal residual disease (MRD), immunophenotype, multiparametric flow cytometry (MFC), relapse

## Abstract

**Simple Summary:**

Leukemia stem cells (LSCs) are rare cell populations present in acute myeloid leukemia (AML) that are resistant to chemotherapy and cause disease relapse in patients even after initial therapy-induced disease remission. Minimal or measurable disease detection (MRD) allows for estimating the residual disease burden after chemotherapy and helps in clinical management of the patient with AML. However, due to current technical limitations, LSCs are not routinely detected as part of MRD assessment, and there is a lack of a standard guideline for quantifying LSCs as part of MRD. This review discusses current research advancements in LSC detection during MRD and outlines future directions for making progress with such research and clinical practice. Such information will be useful to clinicians and biomedical researchers who are trying to advance the field, as well as to the broader research community seeking insight into the pathobiology of and clinical management strategies for AML.

**Abstract:**

Acute myeloid leukemia (AML) is a hematological malignancy characterized by an abundance of incompletely matured or immature clonally derived hematopoietic precursors called leukemic blasts. Rare leukemia stem cells (LSCs) that can self-renew as well as give rise to leukemic progenitors comprising the bulk of leukemic blasts are considered the cellular reservoir of disease initiation and maintenance. LSCs are widely thought to be relatively resistant as well as adaptive to chemotherapy and can cause disease relapse. Therefore, it is imperative to understand the molecular bases of LSC forms and functions during different stages of disease progression, so we can more accurately identify these cells and design therapies to target them. Irrespective of the morphological, cytogenetic, and cellular heterogeneity of AML, the uniform, singularly important and independently significant prognosticator of disease response to therapy and patient outcome is measurable or minimal residual disease (MRD) detection, defined by residual disease detection below the morphology-based 5% blast threshold. The importance of LSC identification and frequency estimation during MRD detection, in order to make MRD more effective in predicting disease relapse and modifying therapeutic regimen is becoming increasingly apparent. This review focuses on summarizing functional and cellular composition-based LSC identification and linking those studies to current techniques of MRD detection to suggest LSC-inclusive MRD detection as well as outline outstanding questions that need to be addressed to improve the future of AML clinical management and treatment outcomes.

## 1. Introduction

Acute myeloid leukemia (AML) is a type of blood cancer, characterized by clonal expansion and accumulation of abnormally or incompletely differentiated immature progenitors or “blasts” of myeloid lineage with associated impairment of normal hematopoiesis, which leads to severe infections, anemia, and hemorrhage. AML is the most common acute leukemia in adults, accounting for 1.3% of new cancer cases in the USA [1]. Although AML can occur at any age, its incidence rate is significantly high in older adults, with a median age of 68 years at diagnosis. Several genetic and environmental risk factors can predispose individuals to the development of AML, and recent advances in genomic sequencing and genetic screening studies have elucidated a few germline predisposing factors to AML development [2]. A history of antecedent hematological disorders, including the myelodysplastic syndromes (MDS) or myeloproliferative neoplasms (MPN), substantially increases the likelihood of disease progression to AML [3,4]. In addition, many patients with nonhematological malignancies later develop therapy-related AML as a result of the cytotoxic chemotherapy administered for their primary malignancy [5].

Standard chemotherapy regimens for AML actively target blasts, leading to initial remission. However, a small number of hematopoietic stem cell (HSC)-derived clones of pre-leukemic stem cells survive cytotoxic chemotherapy. Early preleukemic mutations confer clonal outgrowth advantages to these cells, and lead to clonal hematopoiesis of indeterminate potential (CHIP) [6,7,8,9]. Accrual of additional mutations in these cells then result in the malignant expansion and accumulation of aberrant progenitors or blasts and transform them into leukemia stem cells (LSCs) [8]. Twenty-five years ago, Lapidot et al., described these LSCs for the first time, by showing that a subset of leukemic cells enriched in the CD34+CD38− phenotype could give rise to leukemia when transplanted in immunocompromised recipient mice [10,11]. Since then, several studies have highlighted the quiescent nature of LSCs, which often avoid eradication using conventional chemotherapy, leading to residual disease that fuels high relapse rates and 5-year survival rates below 15% for AML patients. Although several surface markers exhibit higher expression on LSCs when compared to normal HSCs, immunophenotypic identification and isolation of LSCs through detection of these surface markers is limited by the high degree of intra- and interpatient heterogeneity [12]. It is therefore imperative to properly identify these cells, to understand the cellular and molecular bases of disease relapse in AML and identify specific therapeutic strategies for targeting LSCs to improve treatment outcome [13,14].

Diagnosis of AML is carried out using morphological assessment of the peripheral blood (PB) or bone marrow (BM), with immunophenotypic identification of ≥20% myeloid blasts, or using identification of pathognomonic karyotypic or molecular aberrations such as t(8;21), inv(16) or t(16;16), t(15;17), etc. In addition, some patients might also present with extramedullary disease, including involvement of the central nervous system (CNS) [15]. In addition to morphological- and immunophenotyping-based assessments, cytogenetic analysis and screening for commonly occurring gene mutations and rearrangements have enabled classification of AML into various subtypes and risk groups with differing prognostic significance, and complete remission (CR), risk of relapse, and overall survival (OS) rates are stratified by cytogenetic and mutational profile [16,17]. In 2010, the European LeukemiaNet (ELN) defined the first genetics-based stratification system for AML, and successively published revised versions where they further refined the risk stratification of three prognostic subgroups—favorable, intermediate, adverse [18]. AML prognostic assessment at diagnosis allows patients with high-risk disease to receive allogeneic stem cell transplantation (allo-SCT) at first remission as part of frontline therapy [19,20], although the indication for allo-SCT in patients with intermediate risk is currently debated [21]. New developments in the area of targeted therapy in conjunction with conventional chemotherapy may further change the prognostic assessment. As an example, the approval of FLT3 inhibitors such as midostaurin or quizartinib may potentially change the risk-stratification associated with FLT3 mutation status, which is the most common mutation (approximately 30% of AML patients harbor a FLT3 mutation) associated with high risk in AML patients [22,23]. Overall, such risk stratification and prognostication of AML patients is imperative for therapeutic tailoring and disease management of AML patients, which aim to prevent disease relapse and improve long-term patient outcome.

Despite these efforts being implemented for improving disease classification and concurrent therapeutic tailoring, AML is characterized by primary chemotherapy resistance and high relapse rates among patients achieving remission, leading to poor long-term OS. Over 50% of adult AML patients who achieve initial CR rates of >80% after initial treatment experience disease relapse due to reemergence of therapy resistant leukemic clones or blasts surviving at levels below standard detection limits (1 in 10^4^ to 10^6^); because of limitations in standard detection limits, routine optical microscopic quantification or morphological assessment of BM aspirate smear or biopsies at CR cannot detect these extremely rare cells that persist at remission [21,24,25]. Detection of this measurable or minimal residual disease (MRD), defined as post-therapy persistence of leukemic cells, is currently one of the most well-established risk factors in AML. Historically, the cutoff for MRD has been 5%, i.e., 5 residual leukemic cells in 100 cells assessed [26]. MRD is critical in prognostic and risk assessment of patient outcome, which guides patient surveillance for incipient relapse and decision-making for therapeutic management of disease. Therefore, as per updated ELN recommendations, high sensitivity in MRD detection below the morphologic CR represents a critical prognostic and risk assessment strategy for clinical management of AML [18,27]. In such efforts, incorporation of strategies to detect LSCs as part of routine MRD detection will be critical to improving its sensitivity and predictive value. In this review, we aim to describe the role of LSCs in AML pathogenesis and relapse, as well as current MRD detection methodologies and restrictions including recent efforts to incorporate LSC detection as part MRD detection, and to provide our perspective on such endeavors.

## 2. LSCs Drive AML Pathogenesis and Relapse

Evidence found in the last two decades support LSCs as the cell of origin for AML. Primitive HSCs harboring mutations and epigenetic aberrations initially expand into preleukemic cells and ultimately transform into LSCs, which can regenerate and maintain the disease as well as form the cellular reservoir of disease relapse post-therapy. Therefore, therapeutic targeting of LSCs to eliminate them and prevent disease relapse is the most promising avenue for achieving a permanent cure for AML.

### 2.1. Stem Cell Origin for AML

Initial evidence from cytogenetic studies on human AML cell lines and patient samples identified abnormal cells that were capable of long-term culture and multilineage differentiation, suggesting that these aberrations were present in stem cells [28,29,30]. AML pathognomonic chromosomal rearrangements were also detected using fluorescence-activated cell sorting (FACS) in immunophenotypically defined HSCs (CD34+CD38−) and hematopoietic progenitor cells (CD34+CD38+) in primary MDS and AML at diagnosis and at relapse [31,32]. Mouse modeling of preleukemia elucidated that preleukemic hematopoietic stem/progenitor cells (HSPCs) were characterized by their myeloid bias and perturbed differentiation, leading to accumulation of immature myeloid or myelomonocytic cells in the BM and PB. Molecular bases of these cellular defects included downregulation of the transcription factor PU.1 [33], overexpression of the MLL-AF9 and CBFβ–SMMHC fusion oncogenes [34,35], and introduction of CEBPα mutations [36]. Furthermore, transcriptional profiling studies of immunophenotypically defined HSCs from MDS and AML versus normal BM showed that MDS/AML HSCs exhibit gene expression profiles resembling normal HSCs [37,38], with dysregulation in various pathways and signaling processes associated with cellular stemness, emphasizing that AML is a stem cell-driven disease [31,39,40]. FACS and targeted sequencing of HSCs from AML patient samples have demonstrated serial accumulation of genetic lesions in HSCs, and the existence of multiple HSC clones containing a partial repertoire of leukemic mutations [41]. Even at remission, residual CD34+ progenitors as well as mature cells isolated from AML samples have been shown to harbor some of the mutations from the original leukemia [42]. This further supports a stem cell origin of AML, as stem cells are long-lived with high proliferative potential, enabling the mutations sufficient time to accumulate and persist at remission. 

### 2.2. LSC Heterogeneity in AML

Not all leukemia cells are equipotent and only distinct subsets of leukemic cells are capable of initiating disease, as demonstrated first in transplantation studies in immunodeficient mice [10,43] and in vitro clonogenicity assays [44]. Serial transplantations and clonal tracing of leukemic cells from AML specimens identified quiescent long-term leukemia-initiating cells (LICs) that could self-renew and give rise to short-term LICs, eventually generating bulk tumor cells with impaired self-renewal and differentiation capabilities [45]. Extended research has identified different immunophenotypic leukemic cell subpopulations with disease-initiating capabilities other than the CD34+CD38− compartment [46,47,48,49]. However, while determining the cellular architecture of human AML, it is important to acknowledge the limitations inherent in these studies using the xenotransplantation system, which does not reflect the physiological status of LSCs in patients and may enrich for non-physiological functional properties or misrepresent the actual LSC frequency. 

### 2.3. Contribution of LSCs to Relapse

LSCs exhibit stem cell characteristics such as quiescence and drug efflux potential that contribute to their resistance to chemotherapy [50,51]. Xenograft studies in mice have shown that certain quiescent human leukemia cells localize in the BM endosteal region, persist in the face of chemotherapeutic insult, and can generate AML upon secondary transplantation [52]. Sequencing studies have demonstrated that HSCs harboring leukemia-associated mutations are still present after standard chemotherapy in AML patients [42,53]. Due to sensitivity limitations in MRD detection, it is currently not possible to detect whether chemotherapy has eliminated every leukemic cell [53,54,55]; therefore, relapse, driven by residual LSCs (dominant or minor subclones), could be because chemotherapy induces resistance-conferring mutations, or selects for clonal evolution of pre-LSCs [56], with more evidence supporting the former hypothesis [57]. However, sequencing of paired diagnosis and relapse samples has also demonstrated the emergence of a dominant subclone at relapse, which was a minor subclone at diagnosis [42]. 

The stem cell origin of AML relapse suggests that eradication of both malignant and premalignant HSPCs is the most promising strategy for achieving lasting cures. An ideal therapeutic target should be present not only on LSC-enriched populations at diagnosis, but also in the relapse setting, and with expression during CR being predictive of relapse. Thus, it is imperative that the MRD detection incorporates measures to identify LSCs at CR with precision and sensitivity to enable appropriate therapeutic design. 

## 3. MRD in AML

Over 80% of adult AML patients achieve initial CR after undergoing high-intensity chemotherapy (cytarabine and daunorubicin-based “7 + 3” induction chemotherapy), and unfit patients (individuals for whom age, comorbidities, or functional status intensify toxicity of high-intensity treatments, outweighing the benefits) are treated with low-intensity chemotherapy (i.e., hypomethylating agents or low-dose cytarabine) [58]. However, at least 50% of these adult patients experience disease relapse after initial treatment and eventually succumb to their disease. Since residual disease is a fraction of total normal cells measured using the same mechanism, individually dispersed residual leukemic cells may fall below the limit of detection in the sample evaluated. The inability to identify these rare cell populations when they fall below the threshold of detection may result in MRD negativity. In such case, these cells persist as a significant threat to the patient—waiting to reemerge to a level detectable using such testing methods—presenting the risk of overt disease recurrence. Thus, relapse is driven by reemergence of therapy resistant leukemic clones or blasts existing at low levels of 1 in 10^4^ to 10^6^ white blood cells (WBCs) at CR [18,21,24]. Nevertheless, detection of MRD is a necessity, and studies have shown a positive correlation between absence of MRD and favorable prognostic outcome and OS [59,60,61,62]. However, the specific measurement mechanism profoundly affects analytical sensitivity, with different methods having several log-fold variations in limits of detection. Due to this dependence on assay sensitivity, the recognition that a negative test does not imply the absence of disease, and the heterogeneous nature of leukemic cells at the genetic and immunophenotypic level, the technology used to assess MRD is highly critical and needs to be tailored based on the sample [63].

### 3.1. Current Mechanisms and Considerations for MRD Detection

While immunophenotyping with multiparametric flow cytometry (MFC) is the most commonly used MRD monitoring tool, measurement of MRD is routinely performed using other methods as well—BM morphology assessment, cytogenetics, fluorescence in situ hybridization (FISH), polymerase chain reaction (PCR), and molecular genetic tests as per ELN guidelines, American Society of Clinical Oncology (ASCO), and other organizations [18,64,65,66]. Combining MFC and molecular studies has significant value leading to highly sensitive, directed MRD assessment [66,67]. 

Cytomorphology: BM biopsies are routinely stained with hematoxylin–eosin or immunohistochemistry reagents and antibodies for more precise cell identification and are microscopically inspected for morphological assessment. With a threshold of 5% blast detection, this is the oldest method of leukemia quantification with wide applicability and remains a standard CR assessment criteria per national and international expert consensus groups [26,68]. 

Multiparametric flow cytometry (MFC): MFC approaches have gained ubiquitous applicability and suitability, alongside other advantages—rapid turnaround times, relative ease of quantification, ability to distinguish live from dead cells, assessment of hemodilution of the source material, and the possibility to identify immunotherapy targets [63]. This immunophenotyping approach to MRD assessment uses a panel of fluorochrome-labeled antibodies to distinguish leukemic blasts from normal myeloid precursors and identify aberrantly expressed antigens on their cell surface, including co-expression of antigens normally found in early or late hematopoietic differentiation, cross-lineage antigen expression, and over- or under-expression of antigens normally expressed on healthy myeloid cells [18]. ELN has recommended cell phenotype marker panel and gating strategy as per flow cytometry operational guidelines. Two primary methods of immunophenotyping interpretation are utilized—leukemia-associated immunophenotype (LAIP) and the different-from-normal approach (DfN)—and ELN recommends an integration of the LAIP/DfN phenotypes in MRD detection, with the recommended threshold for positivity at ≤10^−4^ [18,69]. The LAIP method identifies one or more leukemia-associated immunophenotypes at diagnosis and is tracked throughout the treatment course. The DfN approach looks for deviant cellular phenotypes compared to typical antigen expression patterns of lineage-committed and maturing cells. While being widely utilized, there are some significant limitations to MFC—(i) MFC assays require extensive analytical experience and expertise and interinstitutional assay standardization. (ii) Not all cases of AML have an abnormal immunophenotype and/or may evolve over time. (iii) Assay sensitivity is subject to patient sample heterogeneity, data analysis typically has subjective elements, and fresh sampling and sampling adequacy is required to yield accurate results [63]. (iv) Finally, customized and standardized premixes of antibodies that can enable sensitive MRD assessment including detection of rare leukemia cells populations are not yet widely available [70].

Polymerase chain reaction (PCR): PCR-based methods, including quantitative PCR (qPCR) and the newer emerging digital droplet PCR (ddPCR) are used for MRD monitoring with a sensitivity of detection of ≤10^−5^. While qPCR has become a standard, ddPCR is more sensitive, reproducible and rapid [71,72], with the limitation of requiring specific assays for detecting individual gene aberration, and validation from complementary DNA assessment [18]. Though PCR is a widely available technique, the clonal heterogeneity of AML with variable mutational burden and cytogenetic abnormalities, albeit containing a relatively stable genome in most cases and with one of lowest tumor mutational burdens of all cancers, complicates the application of PCR. Available PCR assays account for only 40–60% of AML cases, and identification of new mutation and assay standardization is ongoing [10], underscoring a need for patient-specific PCR panels. Importantly, PCR assays have been developed for three AML phenotypes: (i) acute promyelocytic leukemia (APL) (fusion gene *PML::RARA)*; (ii) patients with *NPM1* and CBF–AML (*RUNX1::RUNX1T1* and *CBFB::MYH11*); and (iii) AML with fusion genes *BCR::ABL1*, *KMT2::MLLT3*, and *DEK::NUP214* [18,25]. Although PCR monitoring of these mutations is preferred over MFC [10], the lack of absolute quantification also complicates standardization of these assays [63].

Next-generation sequencing (NGS): NGS, a high throughput methodology with an ability to identify large numbers of genes at increased depth of sequencing, and to quantify RNA/DNA gene expression and detection of variants at increased resolution, has transformational utilities in AML leveraging over PCR [63]. This technique includes whole genome sequencing, whole exome sequencing, and targeted gene sequencing [54,66,73]. NGS has become a widely available oncology product available at individual hospitals and centralized institutions, simplifying the clinician’s assessment burden to a single panel covering multiple mutations [63]. NGS-based MRD detection panels are recommended to include *FLT3-ITD*, *FLT3-TKD*, *KIT*, *RAS* signaling pathway genes, as well as targeted therapy markers such as *FLT3* or *IDH1/IDH2* [18]. The proposed detection threshold for all molecular studies is 10^−4^ or lower [18]. High expense and extensive resource requirements remain significant barriers to the use of NGS for MRD assessment [74,75]. Another key limitation involving AML biology that limits the use of current NGS approaches as standard for MRD detection, and is consistent with other techniques is that AML represents a heterogeneous disease lacking a unifying definition for a mutational driver, has low/variable mutational burden and large-scale genomic aberrations with limited single point mutations [63]. 

Source material for MRD detection: In spite of it not being a technical or mechanistic consideration, one critical aspect of MRD testing is that it involves invasive patient testing, and several studies have advocated the use of PB instead of BM biopsy samples to ease the sampling burden and associated expense [76,77]. PB MFC has the capability to capture 83% of MRD-positive patients with a specificity of 95% [77]. Additionally, ease of sample access adds to a higher sampling frequency, with potential to mitigate the lower sensitivity achieved using PB sampling compared to BM sampling. 

**Figure 1:**
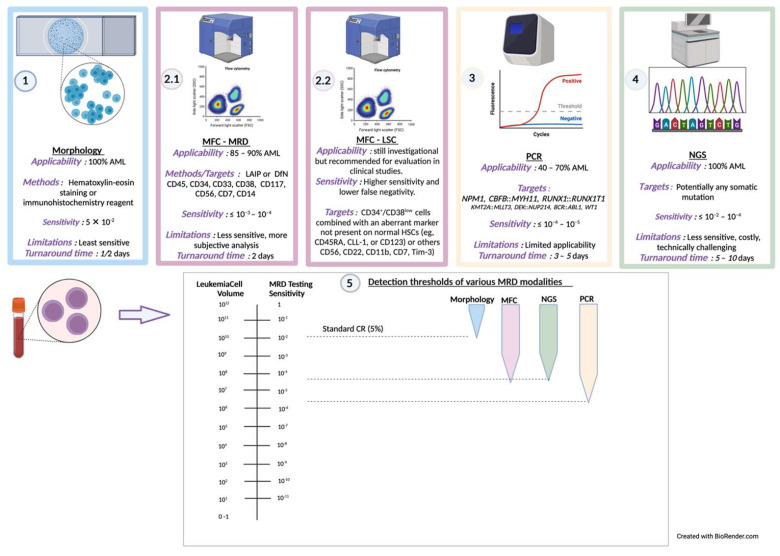
A schematic illustration and description of the clinical MRD detection methods. Panels: 1. Morphology, 2. MFC, 3. PCR, and 4. NGS [18]. Panel 2.2 also demonstrates the LSC investigational detection methods recommended for evaluation in clinical studies [70]. Panel 5 describes the detection threshold of the various detection methods, and is inspired by [84] (Figure 1).

Figure 1 summarizes the clinical application of MRD detection methodologies and the detection thresholds [18] alongside MFC-based LSC detection [70], which is discussed in the next section. 

### 3.2. Prognostic Significance of MRD

Considering the evolving molecular marker dynamics in course of AML diagnosis, therapy and relapse, as per ELN consensus recommendation, leukemic blast burden should be quantified during the time of disease diagnosis, and MRD should be monitored after two cycles of standard or consolidation chemotherapy and at the end of therapy [18]. However, there is variability associated with the frequency of MRD monitoring. In patients with well-characterized AML phenotypes such as APL, CBF, and *NPM1*, follow-up testing is recommended every 3 months using BM sampling in the first 2 years after therapy completion, or every 4–6 weeks using PB [18]. For unfit patients who were treated with low-intensity therapy, the recommendation is to monitor the BM for MRD using molecular markers detected at diagnosis every three treatment cycles, followed by monitoring in the PB every 1–3 months. Some recommendations suggest that detection of markers such as fusion genes of CBF in AML warrant more frequent MRD monitoring to allow for prompt intervention [78,79,80,81,82,83]. MRD monitoring at initial therapy and monitoring at first CR and/or during allo-SCT provide valuable insights into predicting prognostic significance and risk stratification of the disease and guide therapeutic management. 

MRD significantly affects the estimated 5-year disease-free survival (DFS) and estimated OS in AML patients [85], and multiple studies have highlighted the value of MRD status as a prognostic marker in AML [57]. In the AML17 trial, patients with NPM1 mutation and MRD-positive CR had a significantly higher risk of relapse (82%) and lower 3-year survival rate (24%) compared to their MRD-negative counterparts. In the RELAZA-2 trial, AML or advanced MDS patients treated with azacitidine upon detection of MRD positivity during the 2-year post-CR timeline (independent of allo-SCT therapy), had 75% OS at 12 months versus 91% OS observed in MRD-negative patients [86]. In a meta-analysis and systematic review of 11,151 patients by Short et al., the DFS and OS in patients without MRD were 64% and 68%, respectively, and almost halfway reduced −25% and 34%, respectively, in those with MRD [59]. However, the prognostic role of MRD in patients receiving therapy for refractory or relapsed AML is unclear [87]. 

MRD assessment in patients treated with low-intensity chemotherapy also demonstrates significant prognostic value. In the phase 3 PETHEMA-FLUGAZA trial, where 283 elderly AML patients were treated with either fludarabine and cytarabine or azacitidine, patients with MRD-positive CR had significantly worse incidence of relapse and relapse-free survival (RFS) when compared to MRD-negative patients at CR [88]. In another study where AML patients were treated with decitabine and venetoclax, MRD-negative patients had improved RFS and OS compared to MRD-positive patients 2 months after treatment initiation [89]. In the QUAZAR AML-001 trial, an improved RFS and OS post oral azacitidine vs. placebo was seen in both MRD-positive and MRD-negative patient groups [90]. However, in the VIALE-A trial, patients undergoing one cycle of venetoclax and azacitidine therapy and achieving MRD-negative CR had a longer duration of event-free survival and OS compared to patients who had detectable MRD at that time point [79]. 

### 3.3. Current State of MRD Guided Therapeutic Tailoring and Outcomes

MRD monitoring informs decision making for administering targeted therapy utilizing current and novel options indicated to improve patient outcomes and plans for transplant. Post-induction MRD assessment stratifies patients according to relapse risk and selection for allo-SCT [54,78,91] or maintenance using administration of hypomethylating agents [86,92,93]. Some novel agents that are being investigated for application in MRD-positive patients include immunotherapies such as gemtuzumab, ozogamicin and targeted treatments (eg., IDH1 inhibitors ivosidenib and enasidenib, and FLT3 inhibitors such as midostaurin, gilteritinib, quizartinib, and sorafenib) [94,95,96]. Other clinical studies are investigating optimal duration of treatment with chemotherapy agents as a response to MRD assessment [97]. 

MRD status has also been used as a guide for the preconditioning of patients with AML selected for allo-SCT to help refine their transplantation and post-transplantation management. In one study, the patients receiving allo-SCT were randomly allocated into two groups, one that received myeloablative conditioning (MAC) and another that received reduced intensity conditioning (RIC) [60]. Of the patients that relapsed, 71% had been MRD-positive at the time of transplant, supporting the presence of MRD-positivity pretransplantation as a negative prognostic indicator [62]. In addition, only MRD-positive patients (but not MRD-negative patients) who were randomly assigned to RIC had a significantly higher cumulative incidence of relapse (CIR) (1-year CIR 58%) compared with those receiving MAC (1-year CIR 14%; *p* < 0.001), and worse OS (3-year OS 43% vs. 61%; *p* = 0.02). On the contrary, MRD assessment-guided course of treatment have sometimes failed to reproducibly increase OS. For example, in *NPM1* mutant AML patients, conditioning the intensity of regimen in MRD-positive patients did not result in increased OS [98]. Similarly, in several other studies of patients with positive MRD assessed using MFC, there was no positive effect on OS after conditioning the intensity of regimen [99,100,101]. 

The value of MRD assessment prior to allo-SCT, in patients with active disease or post morphologic CR, has been critically established towards prognosis. Araki et al., demonstrated that the 3-year OS and relapse rates were similar in patients transplanted during active disease (23% vs. 26%) versus MRD-positive CR (65% vs. 67%, respectively). In comparison, patient outcomes were improved in patients in MRD-negative CR with 3-year OS at 73%, and relapse rate at 22% [60]. Walter et al., studied 283 patients receiving allo-SCT at first CR (CR1) and second CR (CR2) using a 10-color MFC and found that 3-year estimates of OS were 73% (MRD-negative) and 32% (MRD-positive) for CR1 patients, while for CR2 patients, the OS was 73% (MRD-negative) and 44% (MRD-positive). Disease relapse rates were 58% (MRD-negative) and 21% (MRD-positive) for CR1 patients, and 68% (MRD-negative) and 19% (MRD-positive) for CR2 patients [102]. In another study of 580 patients, MRD status at allo-SCT at CR1 or CR2 remained an independent poor prognostic marker regardless of remission status [103]. In a large study of 1042 patients, the investigators identified high (40%) 2-year relapse rates in MRD-positive vs. MRD-negative (24%) patients undergoing transplantations at CR2 [104]. These studies demonstrate the importance of MRD-guided strategies for reduction of disease burden prior to allo-HSCT, including in MRD-positive states, to achieve long-term survival. In patients with high or adverse-risk AML, use of hypomethylating agents azacytidine and decitabine, and FLT3 inhibitors such as gilteritinib, quizartinib, and sorafenib may be beneficial to lower relapse rates post-transplantation [22,93,105,106,107,108].

MRD-driven relapse is a key concern, and its assessment may impact the preemptive treatment choice in patients after initial chemotherapy. In one study on patients with *NPM1* mutant AML of intermediate risk, favorable outcomes were observed with high dose induction chemotherapy or azacytidine treatment (with or without subsequent allo-SCT), and the 2-year OS was far superior (>80%) for patients who received this treatment preemptively in comparison to patients who received treatment at hematologic relapse [109]. Studies suggest that the application of MRD status for transplant may be most useful in patients with intermediate-risk AML. In the GIMEMA AML1310 trial, a cohort of MRD-positive intermediate-risk AML patients underwent transplantation and experienced favorable outcomes [110]. MRD-guided allo-SCT may improve the DFS and OS in AML patients with non-favorable risk post-induction therapy [91]. Specifically, patients with NPM1 mutation demonstrated higher benefit from receiving transplantation if found to be MRD-positive [78]. Assessment of MRD for application of allo-SCT may be beneficial in MRD-positive intermediate-risk AML and possibly even favorable-risk AML patients [111]. In the AML05 trial, patients with favorable risk t(8;21) AML and persistent MRD who received allo-SCT after the second consolidation therapy had lower CIR and improved DFS compared to MRD-positive patients who received chemotherapy alone [112].

MRD not only provides critical prognostic information enabling risk stratification and therapeutic tailoring in AML but may also be used as a surrogate endpoint for testing drug efficacy, a much faster treatment efficacy assessment measurement compared to monitoring OS. Some of the clinical trials using MRD assessment as an endpoint to evaluate drug efficacy are listed in Table 1. 

## 4. LSC Detection as a Measure for Improving MRD Sensitivity

A significant percentage (20–70%) of AML patients with low/negative MRD levels suffer from disease relapse [119] because traditional MFC methods have the limitation of providing false-negative results at a rate of 13–30% in AML patients. Possible reasons include low sensitivity of traditional methods that cannot detect persisting rare chemoresistant LSCs, immunophenotypic heterogeneity of different AML subtypes [120], variation in antigen expression post-therapy [121], etc. However, recent research advances have suggested that detection of LSCs using MFC confers improved sensitivity and predictive value to MRD detection.

Zeijlemaker et al. [122] designed an eight-color one-tube assay for LSC detection in the CD34+CD38− cell subsets in AML. Using this technique, they were able to detect LSCs using the least possible amounts of BM, both at the time of diagnosis and follow-up, thereby including initially low-frequency populations emerging under therapy pressure. Moreover, this method of LSC detection is optimized for multi-institutional studies. Recently, Li et al. [70] utilized this assay in comparison with the traditional MFC method to assess differences in sensitivity, prediction of CIR, and the time from positive MRD to hematological relapse in AML patients who had received allo-SCT and were randomized into training and validation cohorts. They showed that the false-negative rate was significantly lower (2.5–3.8%) for the CD34+CD38− LSC-based MRD assessment method when compared to traditional MFC methods. Saygin et al., showed that LSC clones present at diagnosis were also detectable in remission samples of patients who achieved morphologic CR. The poor outcome of AML patients was associated with high CD34+CD38− cell frequency, characteristic of LSC, and may inform prognosis [103]. In a separate study, Kamel et al., highlighted that immunophenotypic LSC detection at diagnosis bears significant association with MRD status at early and late postinduction time points and can prognosticate patient outcome [123]. Persistent LSC clones observed during diagnosis may emerge as dominant clones during relapse, or potentially as preleukemic triggering clones via acquisition of new mutations, thus re-emerging as new subclonal populations [57,124]. Therefore, monitoring LSC immunophenotypic and frequency evolution as part of MRD assessment may provide a glimpse on new therapeutic modalities that target this cell population and potentially prevent relapse. Although current MRD monitoring techniques lack the ability to track clonal evolution across rare cell phenotypes such as LSCs, which are challenging to detect and characterize, it is reasonable to hypothesize that LSCs would often be enriched at the time of assessment of MRD [125,126] and therefore must be made a standard assessment criteria for MRD detection. 

MFC identification of LSCs using validated surface markers could be an easy, fast, and readily applicable process across existing clinical settings [20]. The CD34+CD38− cells enriched with LSCs can be further parsed using markers such as CD123, CD99, TIM3, CD7, CD11b, CD22, CD56, and CLL-1 to sensitively identify these highly evolving cells [125,127]. Multiple additional markers utilized to improve detection capability and sensitivity are a subject of research, requiring further assessment and validation using a wide variety of patient samples of different cytogenetic and genomic subtypes [128]. MFC assessment of LSC at MRD is being considered during decision-making related to allo-SCT [125,127]. In support of this, Li et al., showed that LSC detection at MRD may predict relapse and OS after allo-SCT in AML patients with higher sensitivity than traditional MFC-based MRD detection [127]. In a recent study of 155 patients with AML by Canali et al., the assessment of LSC at MRD at postinduction (alongside bulk MRD) showed an independent prognostic impact of LSC in predicting survival outcome [129]. In recent times, increasingly intensifying interest and research in this area emphasizes the importance of LSC quantification in conjunction with bulk MRD assessment as a critical prognostic indicator in AML, and outcomes of these efforts would likely impact the designing of LSC-directed therapies in AML, which will significantly improve treatment outcomes and have lasting effects in patients. 

## 5. Conclusions and Future Directions

In recent years, significant progress has been made towards understanding the pathobiology of AML, specifically in terms of elucidating its genetic and genomic heterogeneity, and these studies have led to the application of novel, targeted therapies and improvement in disease management. As more novel therapeutic agents are being applied in the MRD-directed setting, it is becoming increasingly critical to focus efforts on improving the sensitivity of identification of the biological variables in MRD assessment and unraveling the heterogeneity associated with early stage and relapse stage AML, among other factors. The majority of the studies discussed in this review highlight an increasing need for making MRD assessment highly sensitive, robust, and uniformly applicable across all types of AML. These evidence-based suggestions include incorporating novel immunophenotypic definitions for detection of chemotherapy-adapted/-resistant rare cell populations such as LSCs. 

The heterogeneous nature of AML hinders the realization of a “one size fits all” assay for detecting MRD. To make matters worse, it is not uncommon to see a change in the dominant resistant clone causing relapse post-chemotherapy. The accuracy with which one can lower the threshold for effectively defining a sample as MRD-positive post-therapy may be limited by the variability in relapse outcomes in patients based on the type of treatment as well as the ability of patient’s immune cells to eliminate the disease or both. Monitoring a patient during and post-treatment is also of prime importance, to be able to tailor a treatment based on the patient’s unique disease and response. Equally important is the sample that is used as input for the MRD assay. Enough care needs to be taken so that the BM or PB samples are not hemodiluted or hypocellular in order to enable accurate assessment of MRD. Current efforts are underway for scoping out alternatives to these source materials. Recent research has demonstrated the use of circulating cell-free DNA that was found to be superior to peripheral blood as a prognostic biomarker in patients with AML/MDS undergoing allo-SCT [130].

**Figure 2:**
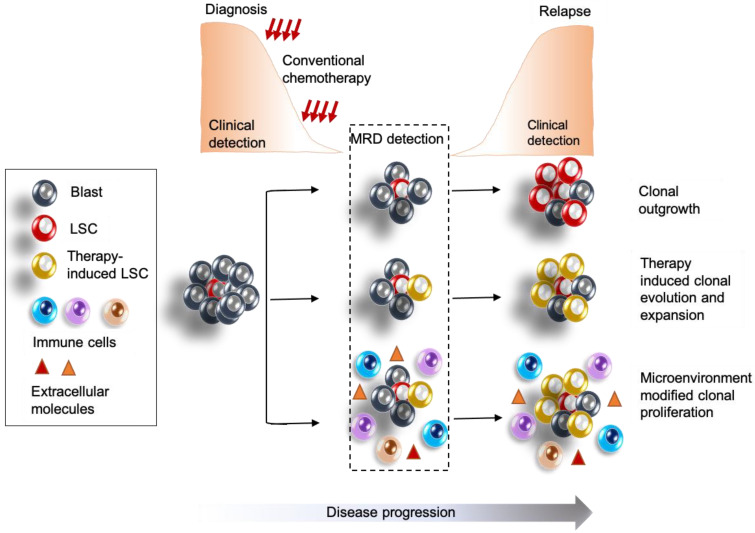
Biological parameters to be considered for improving current status of MRD detection. MRD needs to detect rare chemoresistant LSCs that may persist below the current detection thresholds, as well as incorporate novel markers (evidence-based) to detect new LSCs or leukemic cells that evolve as a response to chemotherapy, and clonally proliferate towards over-disease recurrence. Cell-extrinsic factors and immune cells of the microenvironment in which these leukemia cells reside should be considered as covariates in MRD assessment.

MRD assessment is undeniably complex and the understanding of LSCs further brings forth multiple questions regarding timing, procedure, methodology, sampling, and others. A universal standard guideline to effectively define MRD positivity is restricted by the transplantation assays required to test the capacity of cells to drive clonal outgrowth due to the time-consuming nature of such assays. Importantly, cells that may display engraftment and leukemia generation ability in mice—a necessary criterion for determining the leukemia-initiating capacity of the LSCs—may not always lead to relapse in patients and may be eradicated by host immune cells in the physiological setting. Traditionally applied cytomorphological or MFC methods of MRD assessment being dependent on subjective interpretation might lead to inability to define a cell type as positive for MRD in the lab, which may retain the potential to re-emerge at a later time in the patient. Many AMLs are CD34−, yet functional assays detect the presence of LSCs. For MFC-based assays for LSC detection, there needs to be evidence-based consensus on the panel of markers that should be assessed for detection of these rare cells. Importantly, LSCs have been shown to evolve in frequency and phenotypic diversity in response to current standard chemotherapy [131], highlighting the importance of designing panels that capture the immunophenotypic evolution of these cells pre- and post-treatment. Additionally, as more and more research highlights the importance of considering AML disease evolution in context of the microenvironment, one needs to address how the immune microenvironment impacts the frequency and function of the leukemic cells including LSCs, and how that could impact MRD assessment and therapeutic designs. For example, a highly inflammatory microenvironment enriched in atypical B cells has been shown to be associated with poor outcome in AML patients [132], and one needs to consider how such components of the microenvironment promote the clonal selection and outgrowth of leukemia cell or LSCs that could be measured at MRD. Figure 2 illustrates some of these biological considerations for MRD detection improvement.

The modus operandi of post-MRD testing needs to be optimized based on individual patients, and such an endeavor relies heavily on effective data collection. One solution is to create a comprehensive database consisting of all known markers for MRD and LSCs, molecular as well as genetic, along with predictions of possible shifts in dominant leukemic clones with resultant change in their marker definitions post-treatment based on information available from clinical trial data. An unsupervised assay to detect MRD may also be beneficial, but it may be limited by the speed at which the assay predicts MRD positivity in a sensitive and accurate manner. Timing of MRD assessment is also critical, as current MRD assessments represent static points in the timeline of disease progression and evolution. Instead, serial assessment of MRD at regular intervals might portray a more effective model for predicting disease course. 

Evidence-based research has led to significant advancements in the clinical management of patients with AML and improved their life- and health-span, but there are still many more miles to go. Overall, as our understanding of the disease biology of AML improves, it will synergize with further developments in technology that aim to improve the efficacy of MRD to make it a deterministic parameter for designing patient-specific therapies and lead to the highest yield in terms of improved patient outcomes, enabling them to live longer, disease-free.

## Figures and Tables

**Table 1:** List of currently ongoing clinical trials using MRD assessment as an endpoint measure to evaluate drug efficacy.

Name of Trial	Description
NCT04168502 [113]	Currently ongoing phase 3 study examining MRD levels in AML patients treated with Gemtuzumab in combination with standard chemotherapy.
NCT04093505 [114]	Investigating the role of Glasdegib and Gemtuzumab Ozogamicin (GO) as maintenance therapy post-transplant and as adjunct to consolidation therapy, respectively, to gain evidence of anti-leukemic activity of GO and Glasdegib in older patients with newly diagnosed AML.
NCT04284787 [115]	Currently recruiting unfit AML patients for a phase 2 study for anti-PD 1 pembrolizumab in combination with azacitidine and venetoclax.
NCT04214249 [116]	Currently recruiting AML patients for a phase 2 study for blockade of PD-1 Pembrolizumab in combination with intensive chemotherapy (cytarabine and idarubicin or daunorubicin) as frontline therapy.
NCT03150004 [117]	Currently recruiting patients with R/R and secondary AML to test the efficacy and pharmacogenomics of salvage CLAG-M (cladribine, cytarabine, mitoxantrone, G-CSF) chemotherapy.
NCT01347996 [118]	Evaluating the effects of remission maintenance therapy using Ceplene/IL-2 in adult patients with AML in CR1 on specific immune system cells (T and NK cells) and prospectively defining markers of immune response that are known to reflect T and NK cell ability to combat AML.
