# Correction: Srinivasan Rajsri et al. Acute Myeloid Leukemia Stem Cells in Minimal/Measurable Residual Disease Detection. *Cancers* 2023, *15*, 2866

**DOI:** 10.3390/cancers16050954

**Published:** 2024-02-27

**Authors:** Kritika Srinivasan Rajsri, Nainita Roy, Sohini Chakraborty

**Affiliations:** 1 Department of Pathology, New York University Grossman School of Medicine, New York, NY 10016, USA; ks3144@nyu.edu (K.S.R.);; 2 Department of Molecular Pathobiology, New York University College of Dentistry, New York, NY 10010, USA

## Missing Citation

In the original publication [1], ref. [84] was not cited. The citation has now been inserted in Figure 1 caption and should read:

84. Hourigan, C.S.; Karp, J.E. Minimal residual disease in acute myeloid leukaemia. *Nat. Rev. Clin. Oncol.* **2013**, *10*, 460–471. https://doi.org/10.1038/nrclinonc.2013.100.

## Figure Legend

In the original publication, there was a mistake in the illustration and legend for Figure 1. The graph at the bottom of the figure was improperly labeled in the X-axis, and the figure legend was not written correctly. The correct illustration and legend appear below.

**Figure 1:**
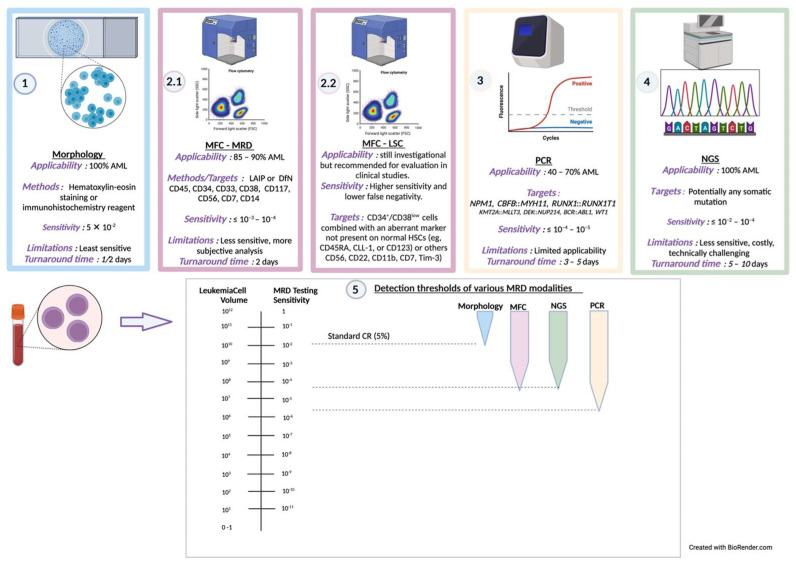
A schematic illustration and description of the clinical MRD detection methods. Panels: 1. Morphology, 2. MFC, 3. PCR, and 4. NGS [18]. Panel 2.2 also demonstrates the LSC investigational detection methods recommended for evaluation in clinical studies [70]. Panel 5 describes the detection threshold of the various detection methods, and is inspired by [84] (Figure 1).

The authors apologize for any inconvenience caused and state that the scientific conclusions are unaffected. This correction was approved by the Academic Editor. The original publication has also been updated.
